# Enhancer‐dependent gene regulation in space, time, and malignancies

**DOI:** 10.1002/ijc.35350

**Published:** 2025-01-31

**Authors:** Belinda Blum, Victoria Dachtler, Angelika Feldmann

**Affiliations:** ^1^ Mechanisms of Genome Control German Cancer Research Center (DKFZ) Heidelberg Germany; ^2^ Faculty of Bioscience Ruprecht‐Karls‐University of Heidelberg Heidelberg Germany

**Keywords:** cancer epigenetics, distal regulatory elements, enhancer hijacking, genome structure, temporal dynamics

## Abstract

Control of cell‐type‐specific gene activation requires the coordinated activity of distal regulatory elements, including enhancers, whose inputs must be temporally integrated. Dysregulation of this regulatory capacity, such as aberrant usage of enhancers, can result in malignant transformation of cells. In this review, we provide an overview of our current understanding of enhancer‐driven gene regulation and discuss how this activity may be integrated across time, followed by epigenetic and structural alterations of enhancers in cancers.

## Graphical abstract

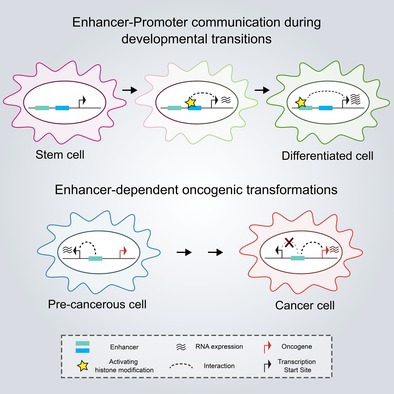

## 1 INTRODUCTION

Correct activation of cell‐type‐specific transcriptional programs requires a tight regulation of gene expression in space and time. This is achieved with the help of remote sequences called distal regulatory elements (DREs), including enhancers, that serve as a platform for the binding of sequence‐specific transcription factors (TFs) and other protein complexes.^1^ From distances of up to several megabases,^2^, ^3^ enhancers are thought to communicate regulatory information by moving into close proximity with their target gene promoters.^4^ In line with the important role of spatial proximity in gene regulation, alterations of enhancer–promoter (E–P) interactions have been linked to various pathologies, especially developmental disorders and cancers.^5^ This review will first describe how enhancers are mapped and identified in the genome using their epigenetic properties. We will then provide an overview of different types of DREs, the dynamics of their interactions with gene promoters, and potential implications for transcription. Finally, we will highlight how E–P interactions and corresponding epigenetic modifications are dysregulated in various cancer types.

## 2 FROM ENHANCER MAPPING TO ENHANCER FUNCTION

Enhancers, as *cis*‐regulatory elements, are traditionally defined by their ability to activate genes independently of their orientation and distance,^6^ a definition that has been in place for more than 40 years, but challenged by recent synthetic genomic studies.^7^ Compared to transcription start sites (TSSs), enhancer location in the genome is more challenging to determine given their often unpredictable position, large distances to target gene promoters, and propensity to skip genes (reviewed in Refs. 4, 8). Molecularly, enhancers are clusters of TF binding sites (TFBSs) and in principle TFs can be used to determine enhancer location. However, given their large number (~1500 in mammalian genomes), enhancer mapping throughout the genome is usually done by methods detecting different enhancer characteristics.

A milestone in enhancer mapping was the detection of characteristic epigenetic signatures, which are still used to distinguish enhancers from promoters.^9^, ^10^ In their landmark studies, Heintzman and colleagues identified high H3K4me3 levels as predictive of gene promoters and H3K4me1 as characteristic of enhancers,^10^ which subsequently led to the identification of cell‐type specific enhancer signatures.^9^ Following this, Rada‐Iglesias et al. were able to distinguish active transcriptional enhancers (H3K4me1+H3K27ac) from those poised for activation later in development (H3K4me1+H3K27me3, “poised enhancers”)^11^ based on the associated histone modifications. Primed enhancers are a third type of enhancers that are neither active nor repressed and are characterized by sole H3K4me1 enrichment.^12^ Finally, polycomb‐mediated H3K27me3 without H3K4me1 characterizes repressed enhancers. Other early detected hallmarks of active enhancers are a high cell‐type‐specific enrichment of the cofactor p300, occupancy by the mediator complex, high chromatin accessibility^9^ and nucleosome depletion.^13^ Consistent with a high occupancy by TFs, active enhancers also display low DNA methylation levels^14^ triggered by a combination of active and passive, TF‐dependent, turnover of DNA methylation.^15^, ^16^ More recently, the discovery of enhancer RNAs (eRNAs) has provided an additional marker for active enhancers,^17^ although not all active enhancers are transcribed.^18^ Despite these characteristic features used for their mapping, not all enhancer signatures follow the same logic. Instead, some active enhancers display high H3K4me3 as opposed to H3K4me1,^19^ or have acetylation marks other than H3K27ac.^20^ Nevertheless, these defined enhancer signatures have been widely used for enhancer mapping in the genome, alongside chromatin accessibility mapping, histone acetyltransferase binding (CBP/p300), or combinations of these. While inherently useful for initial enhancer mapping, all of these methods have limitations. For instance, measuring accessibility provides the fastest route to detect enhancer sites, yet has the disadvantage of not distinguishing between “active” and “inactive” enhancers. Indeed, enhancer activity is detectable in reporter assays for both accessible and inaccessible distal sites,^21^ and polycomb‐occupied regions in the genome can also display some degree of activity.^22^ While the presence of H3K27ac usually indicates enhancer activity, its relative levels are not necessarily predictive of the strength of a given enhancer. Indeed, high H3K27ac levels are associated with both high levels of cell‐type‐specific TFBSs, as well as with weaker activating TFBSs, the latter displaying a lower activity in STARR‐Seq.^21^ Therefore, chromatin features alone may not be sufficient to identify these elements.

Since enhancers can be located at large linear distances from their target genes and also skip genes, functional confirmation of enhancer activity at any given regulatory site remains a complicated task. Assessing enhancer activity usually involves their perturbation by either CRISPR/Cas9‐mediated deletion, silencing, activation,^23^, ^24^, ^25^ or ectopic massive parallel reporter assays (MPRAs), such as STARR‐Seq,^26^ in which an enhancer is placed at the end of a reporter gene to drive its own transcription. CRISPR‐mediated silencing (CRISPRi) and activation (CRISPRa) use repressors or activators (usually KRAB and VP16/64) coupled to catalytically dead Cas9 to silence enhancers. CRISPRi/a‐based approaches and large‐scale reporter assays allow for a higher throughput of tested sequences. However, CRISPRi‐mediated silencing may have the undesired effect of repressing the associated gene promoters in *trans* through interaction with the targeted enhancer. While reporter assays are limited to determining the theoretical ability of an enhancer to activate transcription without taking the chromatin context into account, their advantage is a high throughput as well as their independence of enhancer redundancy, so that enhancers such as “shadow‐enhancers”^27^ remain detectable. Enhancer deletion,^28^, ^29^ on the other hand, allows for an investigation of enhancer activity in their native context but can have undesired structural effects and be difficult to scale up appropriately. Most of our information regarding enhancer function stems from a combination of these methods. These experiments suggest various classifications of enhancer subtypes and have challenged our perception of what constitutes an enhancer. Based on our current understanding of the rules underlying enhancer activity, two groups have recently developed functional synthetic enhancers using computational deep learning methods.^30^, ^31^ These landmark studies propose a new definition, suggesting that an enhancer is first and foremost an element with a lack of repressive DNA‐binding motifs, followed by an enrichment of a small number of cell‐type‐specific “master” TF binding sites.^31^

## 3 FROM ENHANCER TYPES TO TRANSCRIPTION‐ENHANCING ELEMENTS

Enhancer classification considers the chromatin type, the type of associated proteins and the quality of their effect on gene activity. Thus, enhancers have been classified based on the type of their activity or biochemical composition,^11^, ^12^, ^21^ co‐factor requirements,^32^ size/strength,^33^ and the role they play in the regulation of specific genes.^27^ For instance, “shadow enhancers” are redundant enhancers that support transcriptional activity in the absence of the main enhancer of a particular gene.^27^ Clusters of strong cell‐type‐specific enhancers have been called “super‐enhancers.”^33^, ^34^ However, their composition has emerged to be more heterogeneous than anticipated. One such example is the locus control region (LCR) of Hb‐a, considered a classical “super‐enhancer.”^35^, ^36^ Recent dissection of this locus, however, puts forward that some of the acetylated sequences act as facilitators without intrinsic enhancer activity, despite being highly enriched for H3K27ac and therefore epigenetically indistinguishable from other enhancers in the enhancer cluster.^35^ This finding cautions the generalization of super‐enhancers as particularly strong regulatory elements and proposes a more conservative classification of super‐enhancers as regions with a high‐density of potentially regulatory elements. In addition to enhancers, increasing evidence suggests that promoters can also function as autonomous enhancers (reviewed in Refs. 37, 38), while enhancers can act as silencers depending on the cell‐type,^39^, ^40^ further expanding the universe of distal gene regulatory sequences.

Apart from enhancers, which can be described as regulatory elements with autonomous activity, a number of supporting sequences that may be biochemically indistinguishable from enhancers but have no autonomous activation function, have been recently described. These sequences include facilitators,^35^ spatial^41^ and temporal^42^ tethering elements, insulators,^43^ and range extenders.^44^

Insulators in vertebrates usually constitute CTCF‐bound sites placed between a promoter and enhancer, thus preventing their regulatory interaction.^45^ Convergent CTCF sites form boundaries of topologically associated domains (TADs) upon cohesin‐mediated loop extrusion, providing a mechanism as to how such insulation may occur.^46^, ^47^, ^48^, ^49^ In contrast to insulation, tethering elements structurally bring enhancers and promoters closer together by scaffolding two sequences that otherwise would be unable to interact with each other due to large distances. In mouse embryonic stem cells (mESCs), this role has been attributed, for example, to “orphan” CpG islands (oCGIs)^41^ in the vicinity of poised enhancers. These tethering elements interact with clusters of CpG‐islands near gene promoters, thereby presumably promoting long‐range gene activation in differentiated cells. Similarly to tethering elements, “range extenders” (REX) are sequences that enable E–P interactions over hundreds of kilobases‐long distances.^44^ The main difference between these two types of elements is that REXs contain sequence‐specific TFBSs, particularly for TFs containing a Lim homeobox (LHX), whereas oCGIs are bound by general CGI‐associated protein complexes, such as polycomb. Otherwise, both elements appear to represent two different ways of ensuring E–P specificity over large distances. “Facilitators” have been recently described as parts of the alpha‐globin super‐enhancer that are biochemically indistinguishable from other regulatory elements within this LCR.^35^ They are required for the full activation of alpha‐globin in a position‐dependent manner and can be replaced by a facilitator from the beta‐globin locus. Finally, “temporal tethering elements” are transiently promoter‐interacting elements that do not necessarily carry active enhancer signatures and act over long ranges.^42^ Detailed analysis of the Hoxb locus during retinoic‐acid mediated differentiation of mESCs suggests that temporal tethering may facilitate promoter interactions with a strong local enhancer by creating close spatial proximity via transient interactions. Both, facilitators and temporal tethering elements, only partially contribute to gene activation, instead affecting promoter interactions with strong enhancers. Since the dynamics of facilitator interactions have not been assessed yet, these elements may function in a similar manner.

These examples highlight the complex landscape of enhancer‐regulating sequences. We will refer to these types of elements collectively as “transcription‐enhancing elements.”

## 4 ENHANCER–PROMOTER COMMUNICATION IN SPACE AND TIME

Another key aspect of our understanding of enhancer biology is determining how they are able to communicate with gene promoters. Information exchange between enhancers and promoters can span megabase‐long distances.^3^, ^50^ This is thought to be achieved by creating close spatial proximity. Such proximity can be established via protein‐dependent physical interactions or via phase separation,^51^ where transcription (co‐)factors can be shared by multiple loci without necessarily bridging them physically. Another recently proposed theoretical model is the TF activity gradient (TAG),^52^ in which TFs that were acetylated at enhancers freely diffuse to nearby promoters. Since acetylation is rapidly removed, the potential of a regulatory effect depends on the time the acetylated TF requires to reach its target promoter, and thus indirectly on the proximity between the two regulatory elements. Finally, indirect interactions may also create proximity, as has been proposed for enhancer “chains,”^53^ where an E–P connection may be established via a third enhancer element connecting with both. These models and underlying mechanisms have been extensively reviewed recently^8^ and it is likely that all of them contribute to enhancer‐driven gene regulation. In this paragraph, we will therefore focus on E–P interactions as defined by proximity ligation assays (Hi‐C and other 3C methods), keeping in mind that other proximity models likely contribute to the full activation of genes.

Contact formation can be regulated by a variety of mechanisms. Those can include transcription co‐factors such as Ldb1,^54^ insulators such as CTCF,^49^, ^55^ looping factors like YY1,^56^, ^57^ the Mediator complex,^28^, ^58^, ^59^ and RNA Polymerase II (PolII).^60^, ^61^ Less specific regulators of physical interactions, given that they are more broadly distributed in the genome, include histone modifying complexes such as polycomb (PRC1 ad PRC2)^12^, ^28^, ^62^, ^63^, ^64^ and trithorax complexes (MLL3/4)^65^ or CXXC‐domain proteins.^28^, ^55^, ^66^, ^67^, ^68^ Finally, there are factors regulating the formation of large topological domains, such as cohesin/CTCF that mediate loop extrusion.^55^, ^66^, ^67^, ^68^

Substantial evidence links E–P interactions and gene activation. For instance, their acquisition correlates with cell‐type‐specific gene expression during ESC differentiation,^69^ erythropoiesis,^70^ mouse brain development,^71^ gene activation in neurons,^72^ and in various cancers.^46^ Further in support of this model, synthetically generated E–P proximity can cause gene activation^54^, ^73^, ^74^ and global prevention of new interactions in cohesin depletion experiments is associated with reduced gene activation.^75^, ^76^ Moreover, a high number of promoter interactions correlates with high transcriptional levels of the associated genes^69^ and is predictive of their transcriptional regulation in the carcinogenesis of colon cancer.^77^ *De novo* formation of such interactions often correlates with “active” histone modifications, such as H3K27ac, and their instructiveness may differ between cell types or along a specific developmental trajectory.^78^ Interestingly, E–P contact frequencies proved useful, but not strong, predictors within enhancer activity models, such as the Activity‐by‐Contact (ABC) model,^79^ which combines accessibility, H3K27ac enrichments and Hi‐C contact frequencies to predict the effect of a given enhancer on transcription of a given gene. Interactions do not have to be exclusive, since several enhancers appear to be able to interact with the same gene,^80^ forming large clusters or “communities” that share specific gene promoters. Such hubs are used during olfactory receptor selection^81^ or mesenchymal stem cell differentiation.^82^ Importantly, enhancers do not always have to directly interact with the target gene, and instead one enhancer^53^ or tethering element^41^ may connect the entire enhancer community to the promoter.

**FIGURE 1:**
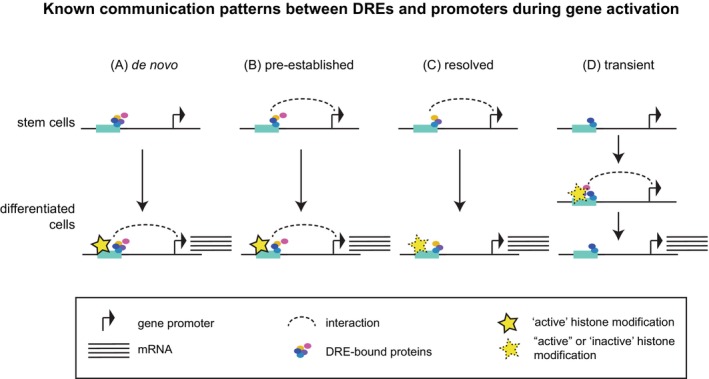
Enhancer–promoter communication patterns during gene activation. Enhancer–promoter interactions can form (A) de novo, (B) be pre‐established, (C) resolved, or (D) transiently formed during normal development.

Despite the evidence of their role in instructing transcription, E–P interactions do not always occur at the exact time point of gene activity (as depicted in Figure 1A), raising the question of whether the timing of such interactions is important. This is especially evident at single‐cell level, as there is cell‐to‐cell variability in interactions during transcriptional bursting.^74^, ^83^, ^84^ Instead, a variety of studies have reported a disconnect between promoter interactions and transcription along developmental trajectories (Figure 1B,C). For instance, physical interactions coinciding with RNA PolII binding can occur hours prior to the activation of the associated genes in *Drosophila* embryonic development,^85^ or be persistent throughout mammalian differentiation (Figure 1B), including cell types in which a particular gene is silent.^12^, ^86^ Other studies identified E–P interactions that are lost upon transcriptional activation.^28^, ^87^ Such interactions typically change their histone modifications from repressive/neutral to activating enhancer signatures, but can also remain without canonical active histone modifications (Figure 1C). Additionally, transient interactions can slow down transcriptional repression^88^ or modulate gene activation^42^ (Figure 1D). More recently, interactions between synchronized promoters have been proposed to be required for appropriate transcriptional activation timing.^89^ Such regulation may be repressive, as is the case for polycomb‐occupied DREs,^12^, ^64^, ^90^, ^91^ and synchronous de‐repression of coregulated genes may be essential for the appropriate timing of their activation.^92^ Taken together, during transcriptional activation, E–P interactions undergo dynamic remodeling. In future studies, it will be interesting to further explore whether and how topological dynamics involving many DREs can affect transcriptional induction and how the information provided by various E–P interactions is integrated at gene promoters.

## 5 ENHANCER TYPES AND INTERACTION TIMING

Our current understanding of how enhancer activities and their communication with gene promoters are integrated is limited. In this paragraph, we will discuss and propose several models of how this may be afforded given different types of regulatory elements as well as distinct timing of their promoter interactions.

To understand the role of temporal variation in contact formation, it is important to consider the possible roles of promoter interactions as well as how they may be related to different types of transcription‐enhancing elements. Interactions may bring regulatory elements together in several different ways. For instance, they can (i) directly juxtapose two regulatory elements, (ii) bring an inherently unsticky regulatory DRE into close proximity to a gene promoter or (iii) bend the DNA to allow for a favorable configuration to occur for another regulatory interaction. Different types of interactions may be used by different types of DREs and may partially dictate the timing when such interactions occur. For instance, facilitating regulatory interactions (ii and iii) may be required to precede E–P interactions to have an impact on gene expression.^42^ A second consideration is that the variability in interaction timing may result from various combinations of a highly specific regulatory mechanism that brings enhancers to promoters at required time points and a general low‐specificity/low‐affinity mechanism in which biochemically compatible elements coalesce.

**FIGURE 2:**
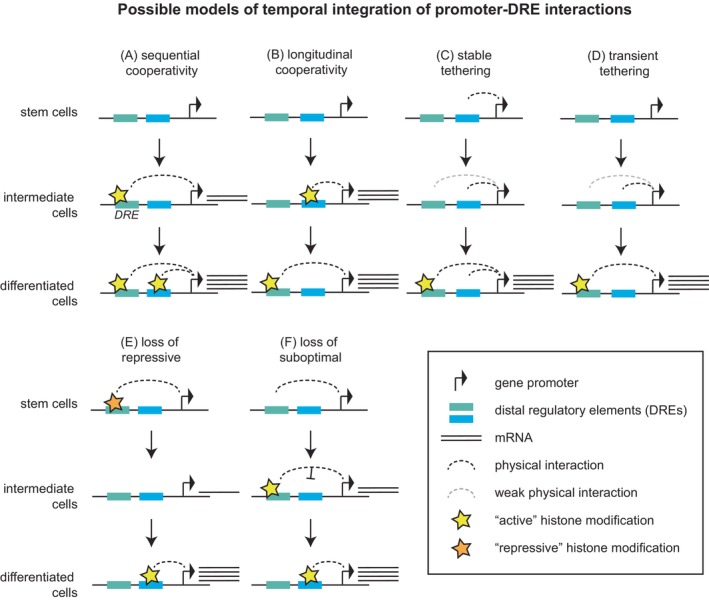
Models of how the temporality of enhancer–promoter interactions may be integrated during gene activation. (A) Sequentially activated DREs may cooperate additively. (B) Sequentially active DREs may cooperate longitudinally. (C,D) Tethering elements form a constitutive (C) or a transient (D) interaction, thereby promoting the interaction with a strong enhancer. (E) Interactions with repressive DREs may have to be lost to afford full activation. (F) Suboptimal DRE interactions may have to be lost to allow for a more activating interaction to occur.

The simplest explanation for the variation in contact timing is the timing of enhancer activity (Figure 2A,B). Assuming an interaction with the promoter is required for transcription, enhancers would have to contact their target gene promoters when they are active themselves, and therefore, the time point of promoter interaction may be dictated by the time point of enhancer activity.^88^ This scenario would comprise pre‐established interactions and sequences of events in which an enhancer becomes activated first, followed by interactions with the associated gene promoter, which then becomes activated itself.^72^ For enhancers that are active in a sequential manner, it is conceivable that they would need to contact their target promoters at consecutive time points to regulate transcription, which would allow them to longitudinally cooperate. Similarly, (temporal) tethering elements, or the first enhancer in a chain,^53^ may have to establish promoter interactions prior to transcriptional activation as well as prior to the activation of associated enhancers to allow for these enhancers to rapidly establish communication with gene promoters once they become activated themselves (Figure 2C,D). These enhancers may be activated after the tethering has occurred and may then take over the interactions with corresponding gene promoters. In principle, such interactions would allow tethering^41^ and facilitating elements^35^ to interact with cognate gene promoters in a transient manner^42^ in‐between regulatory interactions, as well as constitutively. Preformed interactions may be required in cases when gene regulation must occur rapidly upon a specific stimulus. Conversely, a loss of interactions with repressive sites or suboptimal DREs may be essential to enable full and strong gene induction (Figure 2E,F). While repressive polycomb interactions may promote appropriate or synchronized transcriptional activation, their prolongation may become inhibitory.^90^, ^91^ Finally, shadow enhancers^27^ may form either constitutive interactions or no interactions at all, until they are required in the absence of the main regulatory enhancer.

Intrinsic E–P specificity may provide another layer that informs the regulatory effect of E–P interactions.^93^, ^94^, ^95^ It is possible that interactions with specific strong regulatory DREs are acquired at specific time points to allow for transcriptional regulation. However, weak unspecific regulatory interactions may also be formed via the association of compatible broadly bound proteins or protein complexes at two genomic sites. One form of such unspecific coalescence may be provided by histone modifications. In support of this possibility, regions with similar histone modifications generally coalesce to form A and B compartments visible in Hi‐C data,^96^ and sites with physical interactions tend to have similar histone modifications.^86^ For interactions regulated by sequence‐specific TFs, coalescence may be simply regulated by the availability of TFs at specific time points and their weak unspecific interactions with each other. Loop extrusion by cohesin/CTCF can be considered another form of unspecific contact regulation, as it forces regulatory regions into proximity that might not actively interact otherwise.^97^ Such passive interactions may nevertheless enable a certain basal level of gene regulation, provided that regulatory elements can exchange information or “communicate” with each other. Consistent with this possibility, transcriptional activity within TADs is generally correlated between genes and regulatory enhancer units appear to be defined by TAD boundaries.^97^ Gene regulation by less sequence‐specific mechanisms, such as loop extrusion, may be of a smaller magnitude than transcriptional regulation by more specific enhancers. This could explain the small global transcriptional downregulation upon cohesin loss in mESCs.^91^

Highly specific E–P interactions and low‐affinity coalescence between regulatory sites may be two complementary mechanisms required at different stages of gene regulation or for distinct loci. Less specific mechanisms could play a more important role after a gene has been activated. At this time‐point, global chromatin restructuring that accompanies cell‐type transitions would be finished and weak interactions within TAD boundaries could be constantly reinstated by loop extrusion. Then, histone modifications,^65^ transcription co‐factors,^58^ or RNA PolII^60^, ^61^ enriched at an active gene promoter would allow for an association with broadly compatible enhancers within the same TAD. Such scenario could explain a general correlation between the number of promoter interactions and transcriptional activity.^69^ Coexistence of these two models would also explain why some sites (specific) can establish physical interactions even over megabase‐long distances, whereas others (less specific) are unable to sustain such interactions upon genome rearrangement.^98^

It is possible that all of these temporal models and their combinations are used during gene activation in different contexts. Distinguishing between them will require time‐resolved studies of enhancer activity and their productive communication with gene promoters.

## 6 ENHANCER DYSREGULATION IN CANCER

Dysregulation of enhancer activity, including E–P interactions, can result in various diseases, most notably developmental disorders and cancers.^5^ Here, we discuss how topological and epigenetic changes in enhancer function can contribute to carcinogenesis.

**FIGURE 3:**
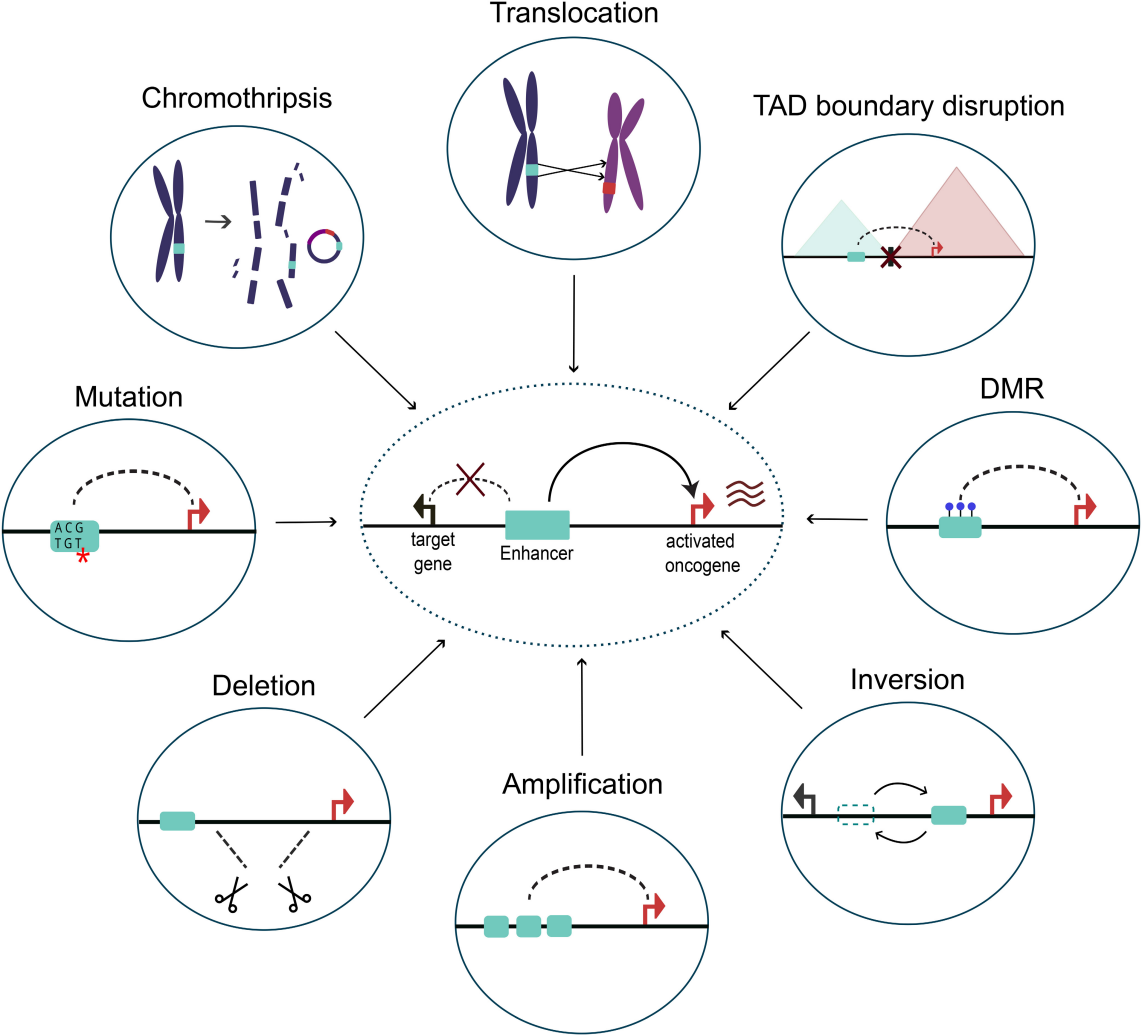
Models of dysregulation in enhancer–promoter communication that can lead to cancer. Several mechanisms contribute to oncogene activation through inappropriate enhancer recruitment, known as enhancer hijacking. Large‐scale genomic events leading to fragmentation and ecDNA (chromothripsis), loss of insulation due to TAD boundary disruption, as well as structural variants, including translocations, deletions, amplifications, or inversions can place enhancers adjacent to oncogenes. In addition, mutations at enhancer sites and epigenetic changes, such as differentially methylated regions (DMR), can lead to aberrant enhancer activity. These changes result in alterations of the chromatin landscape and thus dysregulated gene expression, which drives disease progression.

Enhancer hijacking occurs when enhancers are used by genes that are not their usual targets (Figure 3). If this results in aberrant activation of oncogenes, such hijacking can lead to tumorigenic cellular transformations making it a widespread phenomenon in cancer.^99^ Typical mechanisms involve structural variants (SVs) bringing the oncogene and a strong enhancer into close proximity of each other. Examples of genomic rearrangements of enhancers can be found in adenoid cystic carcinoma (ACC), where a super‐enhancer translocates close to the *MYB* locus, establishing a positive feedback loop that sustains MYB overexpression^100^ and acute myeloid leukemia (AML), where an inversion repositions the GATA2 enhancer near the oncogene EVI1.^101^ Translocations of oncogenes have also been observed for pediatric cancers with very few recurrent genomic drivers, such as medulloblastoma, where *GFI1* and *GFI1B* are brought into the vicinity of active enhancer‐enriched regions resulting in their overexpression.^102^ Neuroblastoma, another childhood cancer with origins in fetal development,^103^ can also be driven by a super‐enhancer translocation into the MYC locus. Apart from being in the vicinity of oncogenes, these enhancers also form physical interactions with MYC, highlighting that oncogene activation can coincide with alterations of the 3D genome structure.^104^ In addition to translocations, enhancer hijacking in neuroblastoma can originate from a focal amplification of active enhancers, which can in turn upregulate MYC expression. A role for E–P loops in carcinogenesis is further highlighted by a recent report that found widespread “neo‐loops” in glioblastomas, whose presence correlated with patient‐specific transcriptional programs.^105^ Determining when such loops first occur in patients may provide an opportunity for early detection of carcinogenesis.

Large‐scale genomic rearrangements can arise during early carcinogenic events, such as chromothripsis, creating new opportunities for E–P communication, for example, due to formation of chimeric extra‐chromosomal DNA (ecDNA).^106^, ^107^ ecDNAs can harbor multiple oncogenes that hijack each other's enhancers to drive their own expression.^108^ This mechanism is used by *CDX2*, a colorectal cancer lineage‐survival oncogene, which was demonstrated to hijack a *MYC* enhancer on the same ecDNA to increase its own expression.^109^

In addition to structural changes, altered enhancer accessibility, generally referred to as “epigenetic” changes, is widespread across different cancer types.^110^ Since enhancer activation correlates with an increase in accessibility, these changes include any variation in enhancer activity, both in *cis* and in *trans*, and are associated with corresponding changes in chromatin modifications. Clusters of active enhancers, or “super‐enhancers,” at key oncogenic loci have been widely observed in the recent years.^34^, ^111^, ^112^ Such enhancer sites can originate *de novo* due to intergenic mutations, as has been observed in T‐cell acute lymphoblastic leukemia (T‐ALL), where recurrent somatic mutations or microinsertions generate new MYB TFBSs, thereby forming enhancers that subsequently drive the activation of TAL1.^113^, ^114^, ^115^ Apart from hyperactivation of enhancers, mutations in distal repressor binding sites can also lead to overexpression of oncogenes. In a recent study in diffuse large B‐cell lymphoma, somatic hypermutations in an intragenic region were shown to alter the binding site of the transcriptional repressor BLIMP1, thereby promoting the overexpression of the oncogene BCL‐6.^116^ Another mechanism of enhancer dysregulation in cancers is super‐enhancer activation by overexpressed TFs.^117^ For instance, while c‐MYC usually binds to E‐box sequences at core promoters, with increasing expression it occupies and activates more distal enhancer sites.^118^ Targeted enhancer activation can be facilitated by lineage‐specific TFs, such as the pioneer TF FOXA1. FOXA1 overexpression has been implicated in genome‐wide enhancer reprogramming and expansion in tumours,^119^ such as in ER^+^ breast cancer, where FOXA1‐mediated activation of a *HIF*‐α super‐enhancer activates a pro‐metastatic transcription program.^120^ Epigenetic reprogramming of enhancers in carcinogenesis can be associated with, or even driven by, DNA hyper‐/hypomethylation leading to differentially methylated regions (DMRs). Consistent with cell‐type‐specific methylation patterns, enhancer methylation is more predictive of tumorigenesis than promoter methylation.^121^ Thereby mutations of enzymes involved in DNA demethylation can result in DNA hypermethylation which correlates with enhancer inactivation, but can also affect DNA structure. For instance, *IDH* mutations in gliomas are associated with hypermethylation at a CTCF‐dependent TAD boundary, coinciding with altered genome structure and enhancer hijacking.^122^ Hypermethylation of enhancers can also result from mutations or inhibition (via mutated IDH)^123^ of the methyl‐cytosine dioxygenase TET2,^124^ promoting the transition to a leukemic transcriptional program in hematopoietic cells.^125^ Hypomethylation of enhancers is also widespread in cancer cells^126^ and preventing demethylation mediated by TET2 condensates has been recently proposed to reduce leukemia growth.^127^

Finally, reactivation of pluripotency and early developmental genes can lead to carcinogenesis by allowing cells to acquire stem‐cell‐like properties.^128^ This can involve repurposing of developmental enhancers through epigenetic reprogramming. The pluripotency gene Sox2 has multiple enhancer sites, and its expression has been shown to be dependent on different ones in different cancer types.^129^, ^130^ In the context of breast and lung cancers, it has been demonstrated that an enhancer site, which is essential for the development of the digestive and respiratory systems, is also activated in a FOXA1‐overexpression‐dependent manner, resulting in aberrant Sox2 expression.^131^ Together with cancer types of early developmental origin, these examples highlight the importance of studying developmental enhancers for our understanding of tumorigenesis.

While epimutations were traditionally considered mere side effects of driver mutations, recently, it began to emerge that they can also occur without known mutagenesis, instead being induced by the cellular microenvironment. For instance, hypoxic conditions in the uterus can promote posterior fossa group A (PFA) ependymoma growth that is associated with H3K27me3 hypomethylation‐based tumorigenesis without known genetic drivers.^132^ This change leads to an altered 3D chromatin landscape characterized by the occurrence of neo‐TADs,^133^ ultra‐long‐range interactions,^134^ possibly also leading to aberrant enhancer usage. Further, as a proof‐of‐principle for epigenetic drivers, a recent *D. melanogaster* model demonstrated that transient suppression of polycomb protein levels can drive irreversible transcriptional changes and carcinogenesis.^135^ Nevertheless, it remains to be shown that such changes can occur naturally.

## 7 CONCLUSION AND OUTLOOK

Despite these examples, supporting evidence for epigenetic mechanisms as a driver in human cancers remains weak, due to heterogeneity, the presence of underlying genomic drivers, and inherent difficulties in identifying the initial causal event. Should they occur, epigenetic drivers are most likely temporally restricted and therefore difficult to assess in retrospective patient studies. Given the widespread role of enhancers in tumor initiation and maintenance, it is likely that any epigenetic mechanism would involve a form of hijacking of enhancer activity. At what stage of carcinogenesis such hijacking occurs remains to be determined. More temporally resolved studies could provide insights into these potential roles. These experiments would have to be carried out at the intersection between carcinogenesis and development, including extensive longitudinal modeling, and possibly single‐cell approaches, such as a recent study identifying the developmental stage of neuroblastoma.^103^

## AUTHOR CONTRIBUTIONS

**Belinda Blum:** Writing – review and editing; conceptualization; investigation; writing – original draft. **Victoria Dachtler:** Conceptualization; investigation; writing – original draft; writing – review and editing; visualization. **Angelika Feldmann:** Conceptualization; investigation; writing – original draft; writing – review and editing; visualization; supervision; resources; funding acquisition.

## CONFLICT OF INTEREST STATEMENT

The authors declare no competing interests.
